# Antimicrobial effect of silver diamine fluoride (SDF) in arresting dentine caries of permanent teeth: A Systematic review

**DOI:** 10.12688/f1000research.158864.1

**Published:** 2024-12-06

**Authors:** Albandri Mohammed Alowid, Mamata Hebbal, Fatimah Salim Almufarji, Ghadeer Ghalab Almutairi, Shahad Saad Alkait, Praveen Jodalli

**Affiliations:** 1 Department of Restorative Dental Sciences, College of Dentistry, Princess Nourah Bint Abdulrahman University, Riyadh, 11671, Saudi Arabia; 2 Department of Preventive Dental Sciences, College of Dentistry, Princess Nourah Bint Abdulrahman University, Riyadh, 11671, Saudi Arabia; 3 Alumni, College of Dentistry, Princess Nourah Bint Abdulrahman University, Riyadh, 11671, Saudi Arabia; 4 Restorative resident, Regional Dental Center in Qassim (RDCQ), Qassim, Saudi Arabia; 5 Public Health Dentistry, Manipal College of Dental Sciences Mangalore, Manipal Academy of Higher Education, Manipal, Karnataka, 576104, India

**Keywords:** : Prevention, Silver diamine fluoride, Remineralization, Antimicrobial effect, Dentine caries

## Abstract

**Background:**

Silver diamine fluoride (SDF) is recognized for its potent antibacterial properties and is commonly used in dentistry to treat carious lesions. This review aims to evaluate the antimicrobial effect of SDF on dentine caries in permanent teeth. The objective is to provide a comprehensive analysis of the existing literature to assess the efficacy of SDF in combating cariogenic flora within dentin lesions.

**Materials and Methods:**

A systematic electronic search was conducted using Google Scholar, Cochrane Library, and PubMed databases to identify relevant studies published between January 2010 and September 2022. The search strategy focused on retrieving in vitro and in vivo studies assessing the antimicrobial effect of SDF on dentinal caries. The inclusion criteria encompassed studies that compared SDF with other antimicrobial agents, such as sodium fluoride varnish, potassium iodide, silver nitrate, chlorhexidine, plasma jet, and deionized water. The Cochrane Collaboration assessment tool was employed to evaluate the risk of bias in the included studies.

**Results:**

Nine studies met the inclusion criteria, consisting of seven in vitro and in situ studies investigating the antimicrobial effect of SDF on dentinal caries, and two in vitro and in vivo studies examining its antibacterial effect on root caries. These studies collectively demonstrated the promising antimicrobial potential of SDF against cariogenic flora present in dentin lesions. However, variations in study design, methodology, and outcome measures were observed across the included studies.

**Conclusion:**

The review underscores the significant antimicrobial efficacy of SDF in combating cariogenic bacteria within dentin lesions of permanent teeth. Despite the promising findings, there remains a lack of comprehensive understanding regarding the precise characteristics and mechanisms underlying the antimicrobial action of SDF, particularly concerning nano-silver. Future research, including long-term clinical trials, is warranted to elucidate optimal dosage regimens and therapeutic approaches for the routine application of SDF in managing dental caries.

## Introduction

Dental caries is the predominant chronic disease affecting both children and adults, leading to significant health burdens globally. Researchers continually strive to find effective management strategies, with minimally invasive techniques emerging as a conservative approach to treating carious teeth. These techniques aim to preserve some of the carious dentin structure before applying a restoration. As part of this approach, antimicrobial agents such as silver diamine fluoride (SDF), sodium hypochlorite, and chlorhexidine are implemented to disinfect the cavity and halt the progression of caries. Silver diamine fluoride, a topical solution comprising fluoride and silver ions, has a long-standing history in medicine and dentistry due to its notable antibacterial properties. Before the advent of antibiotics and amid rising antimicrobial resistance, silver compounds were extensively used for their therapeutic attributes. Today, silver is once again esteemed as an antimicrobial agent for its broad-spectrum activity, low toxicity, and lack of bacterial cross-resistance, particularly within the field of dentistry. Since the 1960s, the combination of silver with fluoride has been promoted as an anti-caries agent, demonstrating significant effectiveness. ^1^

SDF is conventionally used at a 38% concentration, although a 12% formulation is also available. However, clinical studies have indicated that the lower concentration is not as efficacious as the 38% solution in arresting dental caries in children. ^2, 3^ It is particularly indicated for patients with cavitated lesions who face medical management challenges, have difficult carious lesions, carry multiple cavitated carious lesions, are at high risk of caries, or have limited access to dental care. ^2^ Its affordability, straightforward application, non-invasive nature, and minimal application time make SDF an optimal solution for quick, cost-effective, and efficient caries prevention and arrest. ^2, 3^

Clinical studies have demonstrated that SDF can prevent and arrest coronal caries in preschool children’s primary teeth and in the permanent teeth of older children. ^4^ SDF has been shown to be a more effective option for caries control than intermediate restorative treatments, such as glass ionomer sealants, in deciduous teeth., ^5^ Oliveira et al. conducted a systematic review and meta-analysis evaluating the effectiveness of SDF in caries prevention for primary dentition, finding that 38% SDF application reduced the dental caries by 77% in treated children compared to untreated ones. ^6^ Moreover, Chibinski et al. reported that SDF is effective compared to fluoride treatments by 89%. ^7^ However, these studies predominantly focus on caries arrest and prevention in primary dentition and do not comprehensively assess the antimicrobial effect of SDF on permanent teeth, particularly regarding dentinal caries arrest. The objective of this systematic review is to determine the antimicrobial effect of silver diamine fluoride on dentine caries in permanent teeth.

Given the rising incidence of dental caries and the need for effective, non-invasive treatments, this systematic review aims to bridge the knowledge gap regarding the antimicrobial effects of SDF on permanent teeth. While substantial evidence supports its efficacy in primary teeth, less is known about its performance in adult dentition, especially in arresting dentinal caries. Understanding this aspect could enhance clinical protocols and offer a broader application of SDF, providing a potent tool in dental public health. This review will critically assess existing studies, aiming to provide a comprehensive evaluation of SDF’s antimicrobial capabilities in permanent teeth, thereby guiding future research and clinical practice.

## Methods

### Search strategy

An electronic search was conducted using the Google Scholar, Cochrane Library, and PubMed databases. All published in vitro and in vivo studies from January 2010 to September 2022 were included. The review question was formulated using the PICO framework: “Does silver diamine fluoride (I) have an antimicrobial effect (O) on dentinal caries in permanent teeth (P)?” The search keywords used were: (permanent teeth) AND (caries arrest) AND ((SDF) OR (silver diamine fluoride)) AND ((antimicrobial effect) OR (microbial reduction)) AND ((dentin caries) OR (dentinal caries)).

### Inclusion and exclusion criteria

The inclusion criteria were:

• In vitro and in vivo studies
• Published in English
• Focus on dentinal caries in permanent teeth
• Examination of antimicrobial effects
• Caries arrest using silver diamine fluoride

The exclusion criteria were:

• Resources other than articles (e.g., books)
• Non-English language publications
• Studies on enamel/cementum caries or primary/bovine teeth
• Studies not mentioning the antimicrobial effect
• Studies not related to caries arrest
• Studies combining silver diamine fluoride with other materials

### Data collection

**Figure 1.**
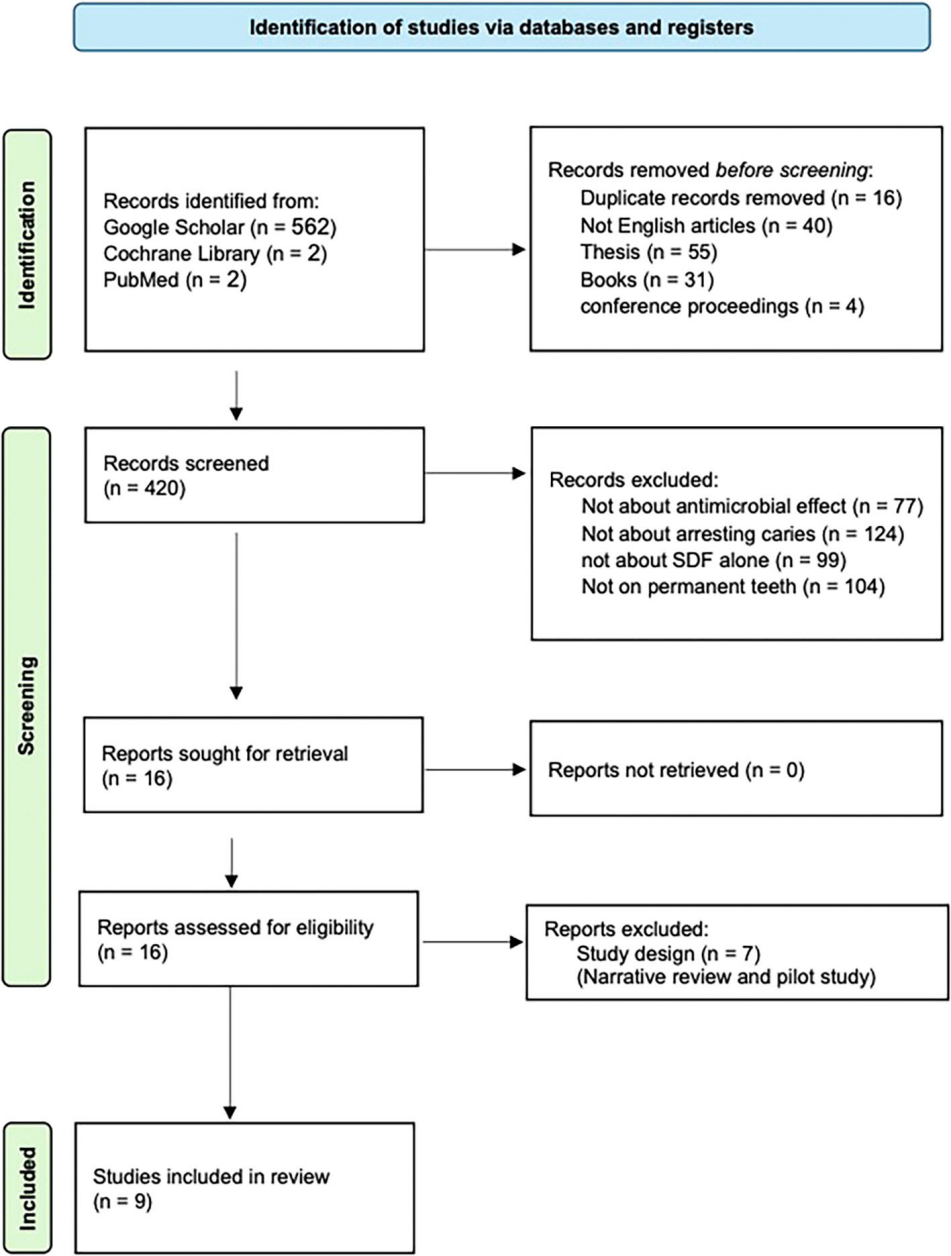
PRISMA flow diagram.

Research articles were collected using Mendeley Desktop 1.19.8 for Windows. The electronic data search and analysis followed the Preferred Reporting Items for Systematic Reviews and Meta-Analyses (PRISMA) guidelines. The search strategy and results are summarized in a PRISMA flow diagram ( Figure 1).

### Screening process

Initially, 566 records were identified and collected from the three databases. After removing 16 duplicates, 40 non-English language articles, 55 theses, 31 books, and 4 conference proceedings, the remaining records were screened. Titles and abstracts were reviewed, and articles meeting the predetermined inclusion criteria were selected. Nine studies were retrieved, and their full texts were screened to ensure they met the eligibility criteria. References from the selected articles were also reviewed to identify additional studies for inclusion.

### Data extraction and analysis

Data extraction and analysis were conducted by two authors independently in multiple steps. Any discrepancies were resolved by a third reviewer, who independently reviewed the articles, and a decision was made based on the majority consensus. The extracted data included study design, sample size, interventions, outcomes, and conclusions.

### Risk of bias assessment

The risk of bias was assessed using the Cochrane Collaboration assessment tool to evaluate the methodological quality of the included studies. Each researcher conducted the risk assessment independently, and any disagreements were discussed and resolved to reach a mutual decision. The risk of bias for each study was documented as low, unclear, or high and summarized in Table 1.

**Table 1.** Risk of bias assessment for each article. 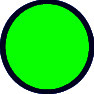
 **: low risk**, 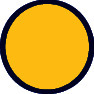 **: unclear risk**, 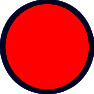 **: high risk**

	Studies	Random sequence generation	Allocation concealment	Blinding of participants and personnel assessment	Blinding of outcome assessment	Incomplete outcome data	Selective reporting	Other bias
1	Abdullah, et al., 2020 ^11^	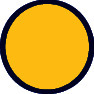	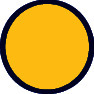	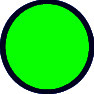	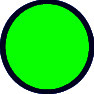	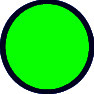	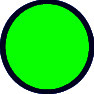	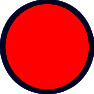
2	Karched et al., 2019 ^16^	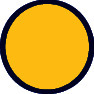	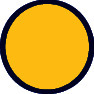	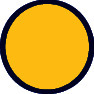	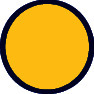	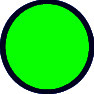	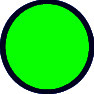	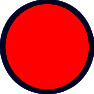
3	Ollie Y. Yu et al., 2018 ^10^	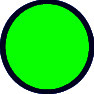	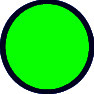	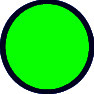	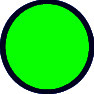	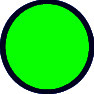	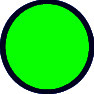	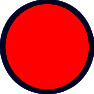
4	Hertel et al., 2018 ^15^	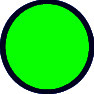	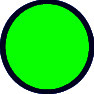	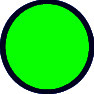	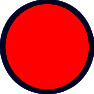	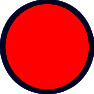	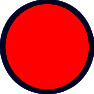	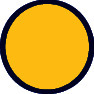
5	Irene Zhao et al., 2017 ^14^	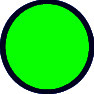	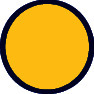	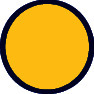	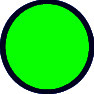	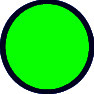	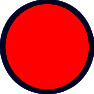	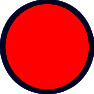
6	Mei et al., 2013 ^8^	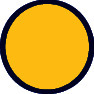	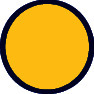	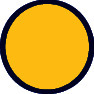	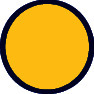	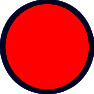	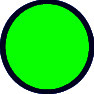	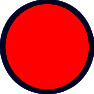
7	Mei et al., 2013 ^12^	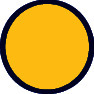	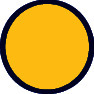	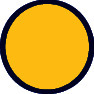	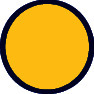	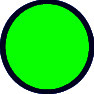	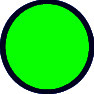	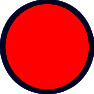
8	Mei et al., 2013 ^13^	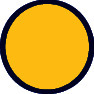	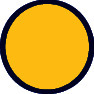	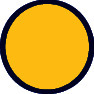	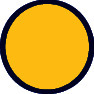	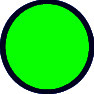	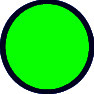	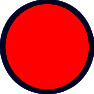
9	CHY et al., 2011 ^9^	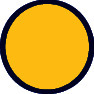	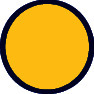	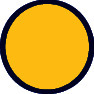	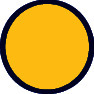	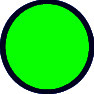	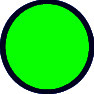	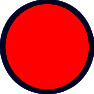

### Data synthesis

A narrative synthesis of the results was performed due to the heterogeneity of the included studies. The antimicrobial effect of SDF on dentinal caries in permanent teeth was evaluated based on the reported outcomes. Meta-analysis was not conducted due to the variability in study designs, sample sizes, and outcome measures. Table 2

**Table 2.** Summary of individual studies.

Study	Intervention and comparator	Objectives	Study design	Brief method	Results and outcome
Mei et al., 2013 ^8^	Test: 38% SDF; Control: Distilled water	Investigate SDF's mechanism in arresting dentin caries	In vitro	12 dentin blocks per group, treated, incubated for 21 days	Higher calcium, phosphorus, and microhardness in test group; SDF inhibits biofilm formation and reduces demineralization (p < 0.05)
Chu et al., 2011 ^9^	Test: SDF; Control: Water	Effects of SDF on dentine carious lesions by S. mutans and A. naeslundii	In vitro	32 demineralized blocks, inoculated, treated	Reduced biofilm counts and increased surface hardness in SDF group (p<0.01, p<0.05)
Yu et al., 2018 ^10^	Test: 38% SDF+5% NaF, SDF, NaF; Control: Water	Antibacterial and remineralizing effects of SDF+NaF	In vitro	104 dentine blocks, treated and assessed for biofilm and lesion depth	SDF+NaF reduced lesion depth significantly; higher antibacterial effect than NaF alone (p<0.001)
Abdullah et al., 2020 ^11^	Test: 38% SDF, 31.3% SDF, SDF+KI, KI; Controls: Water, Chlorhexidine	Evaluate antimicrobial efficacy of different SDF materials	In-situ	90 biofilms from 5 participants, treated with different volumes of SDF	Significant decrease in viable bacteria with 38% SDF (p<0.05); KI addition had no extra effect
Mei et al., 2013 ^12^	Test: 38% SDF; Control: Water	Antimicrobial effect on S. mutans and L. acidophilus	In vitro	30 dentine blocks, inoculated, treated	Significant reduction in CFU counts and higher dead/live bacteria ratio in SDF group (p<0.01, p=0.03)
Mei et al., 2013 ^13^	Test: 38% SDF, 10% NaF, 42% Silver nitrate; Control: Water	Inhibitory effects on demineralized dentin	In vitro	18 demineralized blocks per group, treated	Less demineralization and preserved collagen in SDF group; lower hydroxyproline release (p<0.01)
Zhao et al., 2017 ^14^	Test: 25% Silver nitrate+5% NaF varnish; Control: SDF, Water	Ability to arrest caries	In vitro	18 demineralized dentine blocks, treated	Exposed collagen in control; lower hydroxyproline release in SF and SDF groups (p<0.05)
Hertel et al., 2018 ^15^	Test: Plasmajet CAP I, CAP II; Controls: CHX, SDF varnish, Blank brush	Bactericidal efficacy in root caries lesions	In vitro	50 dentine samples with artificial RCLs, treated	All agents reduced CFU counts; SDF had the highest reduction (p≤0.01)
Karched et al., 2019 ^16^	Test: SDF, SDF+KI; Controls: CHX, Sterile saline	Effect on bacteria in deep carious lesions	In vivo	5 subjects with 5 carious lesions each, treated	Significant reduction in CFU counts for all treatments; SDF most effective (p<0.05)

### Ethical considerations

As this study is a systematic review, it does not involve direct human or animal subjects, and thus, ethical approval was not required. However, all included studies were checked for their ethical approvals and declarations of informed consent.

## Results

The primary objective of this systematic review was to determine the antimicrobial effect of silver diamine fluoride (SDF) on dentinal carious lesions in permanent teeth. The results are presented in two stages: in vivo and in vitro studies, with a summary of the information, reviewers’ assessments, and critical appraisals. Additionally, we investigated whether SDF behaves differently on occlusal dentinal caries compared to root dentinal caries.

### In vivo and in vitro studies

Seven clinical studies were retrieved to investigate the effect of SDF treatment on dentinal caries in permanent teeth.

Mei et al., 2013 evaluated the effect of 38% SDF on cariogenic biofilms and dentin carious lesions using five common cariogenic bacteria. Twelve dentin blocks were treated with SDF, and 12 with water, then incubated for 21 days. The results showed reduced bacterial growth and improved tooth mineral content in the SDF group compared to the control group (p < 0.01). ^8^

In 2011, Chu et al. conducted a study to examine the effects of SDF on dentine caries induced by Streptococcus mutans and Actinomyces naeslundii using artificially demineralized human dentine blocks. The results indicated reduced biofilm counts in the SDF group compared to the control (p < 0.01). Surfaces of carious lesions due to S. mutans were harder (p < 0.05) in the SDF group, while calcium and phosphate content also showed significant reductions after SDF treatment (p < 0.05). These findings suggest that SDF has antimicrobial activity against cariogenic biofilms of both bacteria and slows down dentine demineralization. ^9^

Yu et al., 2018 conducted a study on the effects of SDF solution followed by NaF varnish in treating dentine caries. They used 104 dentine blocks from human third molars, with different groups receiving various treatments and assessments for demineralization and biofilm. The results indicated that the combination of SDF and NaF had a more significant effect on lesion depth compared to individual treatments, showing higher antibacterial effects as well. ^10^

Abdullah et al., 2020 conducted a study to assess the antimicrobial effectiveness of various SDF materials. They used in-situ biofilms from five participants and allowed them to grow for 6 hours. The study compared the anti-biofilm efficacy of different concentrations of SDF, as well as combinations with potassium iodide. The results indicated that all samples treated with 38% SDF showed a significant decrease in viable bacteria compared to the negative control (sterile distilled water). There was also no additional antibacterial effect observed when KI was supplemented with SDF. ^11^

Mei et al., 2013 conducted an in vitro study to investigate the antimicrobial effect of SDF on Streptococcus mutans and Lactobacillus acidophilus co-cultured dual-species biofilm and dentine caries lesions. Dentine blocks from human third molars were inoculated with bacteria, divided into test and control groups, treated with SDF or water, and incubated for 7 days at 37°C. The results showed a significant reduction in CFU counts of both bacteria in the SDF group compared to the control group. SEM images indicated that more bacteria were dead in the SDF group than in the control group. CLSM images also demonstrated a higher dead-to-live ratio after SDF application compared to water. FTIR analysis revealed differences between the log Amide I:HPO42- ratio of the two groups, as did gold-labeling density. ^12^

Mei et al. (2013) further investigated the antagonistic impact of 38% SDF on demineralized dentin. The experiment included 18 blocks of demineralized human dentine that were divided into four groups: one that received a 38% SDF solution, another that received a 10% sodium fluoride solution, a fourth that received a 42% silver nitrate solution, and the control group received deionized water. In the SDF group, smooth, unexposed collagen fibers of dentin were observed. Some collagen fibers were exposed and relatively rough in the sodium fluoride group. In contrast, demineralization resulted in evident exposure of collagen fibers in the silver nitrate and water groups. The mean lesion depth of the silver nitrate and water groups were 259 ± 42 mm and 265 ± 40 mm, respectively, which were significantly higher than those of the SDF and sodium fluoride groups, which were 182 ± 32 mm and 204 ± 26 mm, respectively. The amount of hydroxyproline (HYP), a non-proteinogenic amino acid, in the remineralization solution was used to measure collagen degradation. In contrast to the water (469 ± 63 mg/mL) and sodium fluoride (189 ± 85 mg/mL) groups, the amounts in the SDF (346 ± 57 mg/mL) and silver nitrate (349 ± 18 mg/mL) groups were noticeably higher. ^13^

Irene Zhao et al. (2017) performed an in vitro investigation to see whether a silver nitrate solution and a sodium fluoride varnish might halt the progression of caries. A total of fifty-four dentine slices taken from human third molars were demineralized and processed. After that, the slices were split into three groups: SF, which consisted of 25% silver nitrate and 5% sodium fluoride varnish; SDF, which was a 38% silver diamine fluoride solution and was used as a positive control; and deionized water, which as a negative control. The water group showed exposed dentine collagen fibres, but the SF and SDF groups showed intact and smooth fibres, according to scanning electron microscopy. Both the SDF and SF groups showed less depth of lesion in typical micro-CT images when compared to the water group. There was a statistically significant difference between the water group’s lesion depth (258 ± 53 μm) and the SDF and SF groups, with the former having a mean lesion depth of 135 ± 24 μm and the latter of 128 ± 19 μm, respectively. Moreover, in comparison to the water group (339 ± 16 μg/ml), the SF and SDF groups had a noticeably reduced concentration of hydroxyproline (HYP) in the remineralization solution (312 ± 11 μg/ml and 317 ± 16 μg/ml, respectively). ^14^

### SDF behavior on occlusal and root dentin caries

Hertel et al.’s 2018 in vitro study aimed to assess the bactericidal efficacy of silver diamine fluoride (SDF), cold atmospheric plasmas (CAPs), and chlorhexidine (CHX) in root caries lesions. They created artificial root carious lesions in 50 human dentin samples, dividing them into four groups: two test groups (plasma jet and dielectric barrier discharge source) and two control groups (CHX and SDF varnishes), with a negative control group treated using a blank micro brush to remove nonadherent biofilm. All applied agents resulted in significantly lower colony-forming unit (CFU) counts compared to the control (p ≤ 0.01). When comparing the four treatment agents, only SDF showed a significant decrease in CFU counts (p = 0.004) compared to the dielectric barrier discharge source. ^15^

On the other hand, Karched et al.’s 2019 in vivo study aimed to determine the antimicrobial efficacy of SDF and SDF with potassium iodide. They conducted their study on five patients, each with at least five lesions. Four different agents (SDF, SDF + potassium iodide, chlorhexidine, and sterile saline) were applied to four carious lesions separately in each patient. The mean inhibition zone of S. mutans CCUG 11877 was 25.7 mm, displaying the highest susceptibility to SDF. In the SDF + KI group, the inhibitory zone decreased to 15.15 mm. The inhibition zone of chlorhexidine was 23 mm, while no inhibition was observed in the negative control saline group. The total viable counts of anaerobes were reduced by over 90% after treatment with SDF or SDF + KI, with complete reduction observed in total viable counts of mitis salivarius-bacitracin (MSB) agar. Treatment with SDF decreased the median CFU counts from 9 × 105 to 1.6 × 102 per mg dentin, while in the SDF + KI group, the counts reduced from 2.9 × 105 to 9.2 × 10 per mg dentin. Treatment with chlorhexidine also significantly reduced the median CFU counts. Lesions treated with sterile saline showed no effect on viable counts. Following treatment with SDF, complete inhibition of bacterial growth on Brucella agar was observed in two out of five subjects, with all subjects treated with SDF showing no bacterial growth on MSB agar plates. Complete inhibition of bacterial growth was evident in four out of five subjects following SDF + KI treatment. ^16^ Neither study mentioned a difference in the mechanism of action or behavior of SDF in the crown or root of the tooth. All studies revealed bacterial inhibition after using SDF in both root and occlusal caries.

## Discussion

SDF has been called silver diamine fluoride, ammoniated silver fluoride, and ammoniacal silver fluoride since 1969. ^1^ The accurate terminology is silver diamine fluoride, which has two ammine groups (NH3), however it is sometimes mispronounced. This led to the term “diamine” being used extensively in both academic and marketing contexts. The common ingredients in SDF are ammonia, silver, and fluoride, though the exact amount of fluoride might fluctuate across brands. ^2, 16^ SDF is effective because it combines fluorides and silver nitrates, which is the composition of the compound. By inhibiting collagenase, reducing dentin collagen matrix, and promoting remineralization, SDF inhibits or prevents dental caries. ^3^ It also has an antibacterial effect against dental caries. Particulate silver ions have several proposed means of microbial death, but the specific mechanisms by which they kill bacteria and fungi remain unclear. Some of these mechanisms involve the capacitive binding of silver ions to anionic components of microbial cell membranes. This can cause cell death, cell motility to be rendered ineffective (in motile bacteria), and leakage of cell contents. ^17^ Additionally, some silver ions may act as toxic poisons, inhibiting metabolic enzymes and blocking electron transport chains. ^18^

Furthermore, Chu et al. suggested in their laboratory study that SDF directly inhibits the growth of Streptococcus mutans or Actinomyces naeslundii mono-species biofilms and promotes acid attack resistance in dentin through the reaction of calcium fluoride with hydroxyapatite. ^9^ The high pH of SDF also negatively affects bacterial viability by disintegrating bacterial cell envelopes through the phosphorous and sulfur components of the bacterial cell wall, combined with the reactivity of silver ions, resulting in an antibacterial effect. ^19^ It has been reported that SDF and the combination of SDF with potassium iodide have eliminated bacteria in mitis salivarius-bacitracin (MSB). ^16^ However, an in-situ study found no additional value in using KI with SDF in terms of antimicrobial effect. ^11^ Similarly, an in vivo study showed a better antibacterial effect of SDF and the combination of SDF with potassium iodide compared to chlorhexidine, although this difference was not statistically significant. ^16^

Several studies have demonstrated the superior antimicrobial effect of SDF compared to chlorhexidine as an antimicrobial agent. ^20, 21^ Abdullah et al. found no significant difference in the residual viable biofilm bacteria between the SDF and CHX groups in situ samples. ^11^ Numerous in vitro and in vivo studies have investigated the use of silver compounds in managing human dental caries in permanent teeth. ^22, 23^ However, to our knowledge, there has not been a systematic review that comprehensively examines the antibacterial effect of SDF in arresting dentin caries on permanent teeth and its mechanism of action in both root and crown caries.

## Conclusion

The review of available studies underscores the promising antimicrobial potential of silver-based formulations, such as SDF, against the cariogenic flora prevalent in dentin lesions. However, there remains a significant gap in our understanding regarding the precise size, concentration, antibacterial mechanisms, and toxicological characteristics of nano-silver. To establish a robust foundation for the regular utilization of silver-based compounds as anti-cariogenic agents, it is imperative to generate conclusive evidence through meticulous standardization of experimental protocols, outcome evaluation criteria, and analyses. Clearly, long-term clinical trials are indispensable to gather substantial evidence for making definitive recommendations. Such trials should aim to address the existing knowledge gaps concerning the optimal dosage and therapeutic regimens for the routine application of SDF against polymicrobial consortia present in caries-affected dentin. Through rigorous scientific inquiry and comprehensive clinical investigations, we can advance our understanding and maximize the efficacy of SDF in combating dental caries effectively.

## Reporting guidelines

Repository: PRISMA checklist for ‘Antimicrobial effect of silver diamine fluoride (SDF) in arresting dentine caries of permanent teeth: A Systematic review’. https://doi.org/10.6084/m9.figshare.27814644. ^24^

Data are available under the terms of the Creative Commons Zero “No rights reserved” data waiver (CC0 1.0 Public domain dedication).

## Data Availability

No data are associated with this article.

## References

[1] Zhao IS Mei ML Burrow MF : Effect of silver diamine fluoride and potassium iodide treatment on secondary caries prevention and tooth discoloration in cervical glass ionomer cement restoration. *Int. J. Mol. Sci.* 2017 Feb 6;18(2):340. 10.3390/ijms18020340 28178188 PMC5343875

[2] Mishra A Sahoo P Ray P : Silver Diamine Fluoride: Journey from Silver bullet to Magic Bullet. *Int. J. Sci. Res.* 2019.

[3] Rosenblatt AT Stamford TC Niederman R : Silver diamine fluoride: a caries “silver-fluoride bullet”. *J. Dent. Res.* 2009 Feb;88(2):116–125. 10.1177/0022034508329406 19278981

[4] Llodra JC Rodriguez A Ferrer B : Efficacy of silver diamine fluoride for caries reduction in primary teeth and first permanent molars of schoolchildren: 36-month clinical trial. *J. Dent. Res.* 2005 Aug;84(8):721–724. 10.1177/154405910508400807 16040729

[5] Chou R Cantor A Zakher B : Preventing dental caries in children< 5 years: systematic review updating USPSTF recommendation. *Pediatrics.* 2013 Aug 1;132(2):332–350. 10.1542/peds.2013-1469 23858419

[6] Oliveira BH Rajendra A Veitz-Keenan A : The effect of silver diamine fluoride in preventing caries in the primary dentition: a systematic review and meta-analysis. *Caries Res.* 2019 Jun 6;53(1):24–32. 10.1159/000488686 29874642 PMC6292783

[7] Chibinski AC Wambier LM Feltrin J : Silver diamine fluoride has efficacy in controlling caries progression in primary teeth: a systematic review and meta-analysis. *Caries Res.* 2017 Oct 4;51(5):527–541. 10.1159/000478668 28972954

[8] Mei ML Li QL Chu CH : Antibacterial effects of silver diamine fluoride on multi-species cariogenic biofilm on caries. *Ann. Clin. Microbiol. Antimicrob.* 2013 Jan;12:1–7. 10.1186/1476-0711-12-4 23442825 PMC3599989

[9] Chu CH Mei LE Seneviratne CJ : Effects of silver diamine fluoride on dentine carious lesions induced by Streptococcus mutans and Actinomyces naeslundii biofilms. *Int. J. Paediatr. Dent.* 2012 Jan;22(1):2–10. 10.1111/j.1365-263X.2011.01149.x 21702854

[10] Ollie YY Zhao IS Mei ML : Caries-arresting effects of silver diamine fluoride and sodium fluoride on dentine caries lesions. *J. Dent.* 2018 Nov 1;78:65–71. 10.1016/j.jdent.2018.08.007 30114443

[11] Abdullah N Al Marzooq F Mohamad S : The antibacterial efficacy of silver diamine fluoride (SDF) is not modulated by potassium iodide (KI) supplements: A study on in-situ plaque biofilms using viability real-time PCR with propidium monoazide. *PLoS One.* 2020 Nov 3;15(11):e0241519. 10.1371/journal.pone.0241519 33141868 PMC7608867

[12] Mei ML Chu CH Low KH : Caries arresting effect of silver diamine fluoride on dentine carious lesion with S. mutans and L. acidophilus dual-species cariogenic biofilm. *Med. Oral Patol. Oral Cir. Bucal.* 2013 Nov;18(6):e824–e831. 10.4317/medoral.18831 23722131 PMC3854072

[13] Mei L Ito L Cao Y : Effect of silver-diamine-fluoride on dentine demineralisation and collagen degradation. *J. Dent. Res.* 2013;41:809–817. 10.1016/j.jdent.2013.06.009 23810851

[14] Zhao IS Mei ML Li QL : Arresting simulated dentine caries with adjunctive application of silver nitrate solution and sodium fluoride varnish: an in vitro study. *Int. Dent. J.* 2017 Aug 1;67(4):206–214. 10.1111/idj.12291 28332192 PMC9378902

[15] Hertel M Schwill-Engelhardt J Gerling T : Antibacterial efficacy of plasma jet, dielectric barrier discharge, chlorhexidine, and silver diamine fluoride varnishes in caries lesions. *Plasma Med.* 2018;8(1):73–82. 10.1615/PlasmaMed.2018024767

[16] Karched M Ali D Ngo H : In vivo antimicrobial activity of silver diammine fluoride on carious lesions in dentin. *J. Oral Sci.* 2019;61(1):19–24. 10.2334/josnusd.17-0366 30726799

[17] Nuvvula S Mallineni SK : Silver diamine fluoride in pediatric dentistry. *Journal of South Asian Association of Pediatric Dentistry.* 2019 Jul;2(2):73–80. 10.5005/jp-journals-10077-3024

[18] Fakhruddin KS Egusa H Ngo HC : Clinical efficacy and the antimicrobial potential of silver formulations in arresting dental caries: a systematic review. *BMC Oral Health.* 2020 Dec;20:1–3. 10.1186/s12903-020-01133-3 PMC7268710 32493272

[19] Morones JR Elechiguerra JL Camacho A : The bactericidal effect of silver nanoparticles. *Nanotechnology.* 2005 Aug 26;16(10):2346–2353. 10.1088/0957-4484/16/10/059 20818017

[20] Hamama HH Yiu CK Burrow MF : Effect of silver diamine fluoride and potassium iodide on residual bacteria in dentinal tubules. *Aust. Dent. J.* 2015 Mar;60(1):80–87. 10.1111/adj.12276 25721282

[21] Besinis A De Peralta T Handy RD : Inhibition of biofilm formation and antibacterial properties of a silver nano-coating on human dentine. *Nanotoxicology.* 2014 Nov 1;8(7):745–754. 10.3109/17435390.2013.825343 23875717

[22] Fakhruddin KS Egusa H Ngo HC : Clinical efficacy and the antimicrobial potential of silver formulations in arresting dental caries: a systematic review. *BMC Oral Health.* 2020 Dec;20:1–3. 10.1186/s12903-020-01133-3 PMC7268710 32493272

[23] Shah S Bhaskar V Venkatraghavan K : Silver diamine fluoride: a review and current applications. *J. Adv. Oral Res.* 2014 Jan;5(1):25–35. 10.1177/2229411220140106

[24] Praveen J : Antimicrobial effect of silver diamine fluoride (SDF) in arresting dentine caries of permanent teeth: A Systematic review.[Dataset]. *figshare.* 2024. 10.6084/m9.figshare.27814644 PMC12246775 40655937

