# Supplementary material for: Antimicrobial effect of silver diamine fluoride (SDF) in arresting dentine caries of permanent teeth: A Systematic review
Source: F1000Res. 2024 Dec 6;13:1495. [Version 1] doi: 10.12688/f1000research.158864.1 (PMC12246775; doi:10.12688/f1000research.158864.1)
Supplement: Peer review history

# Reviewer report: Reviewer response for version 1

**DOI:** 10.5256/f1000research.174511.r364146

**Published:** 2025-02-07

**Authors:** Kiran Iyer

**Affiliations:** 1 Department of Public Health, College of Dentistry, King Saud bin Abdulaziz University for Health Sciences, King Abdullah International Medical Research Center, Ministry of National Guard Health Affairs, Riyadh, Saudi Arabia

Aim: This review aimed to evaluate SDF’s antimicrobial capabilities in permanent teeth comprehensively, guiding future research and clinical practice.

 Search: In total 9 articles satisfied the final inclusion and exclusion criteria and were included for final review.

 The PICO-based search strategy has been found to include appropriate keywords.

 RoB: The risk of bias was assessed using the Cochrane Collaboration assessment tool to evaluate the methodological quality of the included studies- appropriate for interventional studies.

 Conclusion: this is very appropriate and in line with findings from studies, there is a need for many other variables to be considered before concluding on the role of nano silvers. Though promising there remains a significant gap in our understanding regarding the precise size, concentration, antibacterial mechanisms, and toxicological characteristics of nano-silver.

Are the rationale for, and objectives of, the Systematic Review clearly stated?

Yes

Is the statistical analysis and its interpretation appropriate?

Not applicable

If this is a Living Systematic Review, is the ‘living’ method appropriate and is the search schedule clearly defined and justified? (‘Living Systematic Review’ or a variation of this term should be included in the title.)

Not applicable

Are sufficient details of the methods and analysis provided to allow replication by others?

Yes

Are the conclusions drawn adequately supported by the results presented in the review?

Yes

Reviewer Expertise:

Oral Epidemiology, Bio-Statistics, Oral Health Utilization, AI in Oral Health

I confirm that I have read this submission and believe that I have an appropriate level of expertise to confirm that it is of an acceptable scientific standard.

# Reviewer report: Reviewer response for version 1

**DOI:** 10.5256/f1000research.174511.r364143

**Published:** 2025-02-07

**Authors:** Nishant Mehta

**Affiliations:** 1 Oral Health Sciences Centre, Postgraduate Institute of Medical Education and Researc, Chandigarh, India

Review summary:

 The topic's area and scope are well justified, given the high burden of dental caries among adults and the limited cost-effective strategies available. The introduction is built upon existing literature and highlights the need for the study. The methods section adequately describes the search strategy, inclusion/exclusion criteria, and risk of bias assessment. Provides valuable insights about future implications and effectively summarizes key findings of the study.

 Recommendations:

 Abstract:

1. Please modify the conclusion to reflect upon the study objectives.

Methods:

1. Please provide the search strategy as a supplementary file.
2. Please remove duplicates from the Exclusion criteria; a) Studies not mentioning the antimicrobial effect and b) Studies not related to caries arrest
3. Please mention if any restrictions on study design were made in the inclusion criteria to limit heterogeneity in included studies
4. Kindly consider registering the review with PROSPERO if the same has not been done.
5. Consider using JBI or QUIN tool for quality assessment of invitro studies.

Results:

1. Kindly concise summary of included studies.  
2. Please explicitly mention variability in the included studies leading to not carrying out pooled analysis.

Discussion:

1. 1 ^st^ paragraph of discussion section gives an elaborated description of SDF mechanism which is beyond scope of this study.
2. Please compare the effectiveness of SDF to other antimicrobial agents.
3. Please elaborate upon study limitations e.g., short follow-up duration in studies, in vitro vs. in vivo differences etc.

Conclusion:

1. Please remove or refine speculative claims about nano-silver unless directly supported by the reviewed studies

Are the rationale for, and objectives of, the Systematic Review clearly stated?

Yes

Is the statistical analysis and its interpretation appropriate?

Yes

If this is a Living Systematic Review, is the ‘living’ method appropriate and is the search schedule clearly defined and justified? (‘Living Systematic Review’ or a variation of this term should be included in the title.)

Not applicable

Are sufficient details of the methods and analysis provided to allow replication by others?

Yes

Are the conclusions drawn adequately supported by the results presented in the review?

Yes

Reviewer Expertise:

Public Health Dentistry & Preventive Dentistry, Tobacco & Oral Microbiome Research, Cancer & Oral Mucositis Research

I confirm that I have read this submission and believe that I have an appropriate level of expertise to confirm that it is of an acceptable scientific standard.
